# Facile Synthesis of Enzyme-Embedded Metal–Organic Frameworks for Size-Selective Biocatalysis in Organic Solvent

**DOI:** 10.3389/fbioe.2020.00714

**Published:** 2020-07-07

**Authors:** Yangxin Wang, Ningning Zhang, Deming Tan, Zhenhui Qi, Changzhu Wu

**Affiliations:** ^1^College of Materials Science and Engineering, Nanjing Tech University, Nanjing, China; ^2^Sino-German Joint Research Lab for Space Biomaterials and Translational Technology, School of Life Sciences, Northwestern Polytechnical University, Xi’an, China; ^3^Institute of Microbiology, Technische Universität Dresden, Dresden, Germany; ^4^Department of Chemistry, Technische Universität Dresden, Dresden, Germany; ^5^Department of Physics, Chemistry and Pharmacy University of Southern Denmark, Odense, Denmark; ^6^Danish Institute for Advanced Study, University of Southern Denmark, Odense, Denmark

**Keywords:** metal–organic frameworks, enzymatic reactions, size-selective catalysis, heterogeneous catalysis, biocatalysis

## Abstract

*In situ* immobilization of enzyme into metal–organic frameworks (MOFs) is performed through a one-step and facile method. *Candida antarctica* lipase B (CalB) is directly embedded in zeolitic imidazolate framework (ZIF)-8 by simply mixing an aqueous solution of 2-methylimidazole and zinc nitrate hexahydrate [Zn(NO_3_)_2_⋅6H_2_O] containing CalB at room temperature. Due to the intrinsic micropores of ZIF-8, the obtained CalB@ZIF composite is successfully applied in size-selective transesterification reaction in organic solvent. CalB@ZIF not only shows much higher catalytic activity but also exhibits higher thermal stability than free CalB. Besides, the robust ZIF-8 shell also offers the hybrid composites excellent reusability.

## Introduction

Enzymatic catalysis is one of the most important catalytic processes used in modern industries to manufacture chemicals, pharmaceuticals, food, and materials for human society. Immobilized enzymes are preferred in the industry since free enzymes are usually vulnerable in non-aqueous media or at elevated temperatures (Sheldon, 2007; Zhang et al., 2015). To date, various kinds of solid materials have been employed as carriers for the immobilization of enzymes, including natural polymers, synthetic polymers, inorganic materials, etc. (Hartmann and Jung, 2010; Datta et al., 2013; Zhou and Hartmann, 2013). The immobilization can be approached by different techniques, e.g., adsorption (Wang et al., 2019), covalent binding (Engstrom et al., 2013), affinity immobilization (Keller et al., 2017), and entrapment (Majewski et al., 2017). Generally, the stability of immobilized enzymes is usually enhanced compared with free enzymes, which thus enables the long-term and repeated usage of the enzymes. The ideal enzyme immobilization method should retain maximal enzyme activity while minimizing mass transfer limitation of substrates/products. However, no existing method has entirely fulfilled these requirements (Gkaniatsou et al., 2017). Therefore, developing novel immobilization matrices and methods still remain of key interest. Moreover, the immobilization of enzymes for size-selective catalysis has been rarely reported, though size-selective catalysis is quite important when a reaction is conducted in a complex system containing substrates with the same or similar reaction groups.

Metal–organic frameworks (MOFs) are an attractive class of porous crystalline solids built from metal ions and organic linkers. Due to the easily tunable chemical and structural properties, MOFs have shown great potential in applications in various areas, including gas adsorption (Xiao et al., 2016), catalysis (Huang et al., 2017; Zhu et al., 2017), and drug delivery (Chen et al., 2017; Wu and Yang, 2017). Recently, the application of MOFs as matrices for immobilizing proteins/enzymes has also emerged (Liang et al., 2020). Enzymes could be immobilized through adsorbing (Liu et al., 2013, 2014, 2015; Zhao et al., 2015; Cao Y. et al., 2016; Lu et al., 2016; Zhang et al., 2017; Samui and Sahu, 2018) or crosslinking (Jung et al., 2011; Shih et al., 2012; Doherty et al., 2013; Patra et al., 2015; Cao S. et al., 2016; Patra et al., 2016; Wang et al., 2016; Wen et al., 2016) onto the surface of MOFs. It was also reported that some enzymes could enter the interior of MOFs and be stabilized in the pores (Lykourinou et al., 2011; Chen et al., 2012a; Deng et al., 2012; Kim et al., 2015), whereby enzymes undergo significant conformational change due to the strong interactions between enzymes and organic linkers in MOFs (Chen et al., 2012b, 2014). Prominent enhancement of stability and/or activity of enzymes was observed when they were immobilized on MOFs, indicating that MOFs were among the promising matrices for immobilizing enzymes (Li et al., 2016a, b; Nadar and Rathod, 2018a).

It should be noted that the abovementioned immobilizing enzymes on/in MOFs are usually realized through at least two steps, namely, preparation of MOFs and immobilization of enzymes. One-step fabrication of enzyme/MOF hybrids would be of great interest for its ease of preparation, which requires that the formation of MOFs should proceed in a fast and mild manner and would preserve the activity of enzymes (Gascon Perez et al., 2017). Zeolitic imidazolate frameworks (ZIFs), especially ZIF-8, which grow under mild biocompatible conditions and feature with exceptional chemical and thermal stability, have been primarily studied for the immobilization of enzymes in a one-pot reaction. Liu et al. did the pioneering work in this field, embedding protein (cytochrome *c*, Cyt *c*) in ZIF-8 in methanol. During the synthesis, polyvinylpyrrolidone (PVP) was utilized for enhancing the dispersion and stabilization of Cyt *c* (Lyu et al., 2014). Besides, the protective effect of ZIF-8 for enzymes/proteins, including urease and horseradish peroxidase (HRP), against polar solvents, high temperature, and trypsin was demonstrated by Falcaro’s group (Liang et al., 2015). Shieh et al. (2015) embedded catalase into ZIF-90, showing that the catalase exhibited hydrogen peroxide degradation even in the presence of protease because the catalase was sheltered and protected by ZIF-90 shell. Ge group prepared a hybrid biocatalyst by embedding glucose oxidase (GOx) into ZIF-8, which was further modified with polydopamine (PDA). This composite can be used repeatedly without obvious activity loss (Wu et al., 2015b). Later, GOx/ZIF-8 composites were reported as chemical sensors (Hou et al., 2015; Wang et al., 2017). Ge group further investigated the protective effect of ZIF-8 for the embedded enzymes in various denaturing organic solvents (Wu et al., 2017). The same group also developed multienzyme-containing ZIF-8 composites by immobilizing different enzymes in ZIF-8 in one-pot in aqueous solution at 25°C. High catalytic efficiency, selectivity, and stability of this hybrid material were displayed due to the existence of ZIF-8 shell (Wu et al., 2015a; Chen et al., 2018). Laccase was also entrapped into ZIF-8, showing increased thermostability, reusability, and storage stability (Patil and Yadav, 2018). Based on these reports, it is demonstrated that embedding enzymes in ZIF-8 can proceed in biocompatible conditions in a facile one-step method, holding considerable potential in developing enzyme/MOF hybrid catalysts.

*Candida antarctica* lipase B is a well-known and easily available lipase, which can catalyze esterification, hydrolysis, and transesterification reaction efficiently. Recently, hybrid materials prepared through embedding CalB and other lipases in ZIF-8 were reported by several research groups (Pitzalis et al., 2018; Cai et al., 2020). Rathod group encapsulated lipase (from *Aspergillus niger* source) in ZIF-8 by mixing an aqueous solution of Zn(NO_3_)_2_ containing lipase with an aqueous solution of 2-methylimidazole (Nadar and Rathod, 2018b). It was found that the ultrasound treatment of lipase before encapsulation increased its activity, probably due to the favorable conformational changes of lipase during sonication. Further, the same group found that the activity of the lipase could be improved in the presence of proline, which was able to maintain active conformation of enzyme and protect active sites under high-temperature conditions (Nadar and Rathod, 2019). Surfactants were also capable to enhance the activity of encapsulated lipase in ZIF-8, as the hydrogen bonding and hydrophobic–hydrophilic interactions between surfactants and lipase were believed to improve the 3D conformation of lipase (Vaidya et al., 2020). It is noticed that these works are mainly focusing on the improvement of catalytic activity of encapsulated CalB or other lipases in ZIF-8 by various methods. Due to the relatively small aperture diameter (3.4 Å) of ZIF-8, assembled ZIF-8 particles have been applied as shells of hybrid catalytic materials for size-selective catalysis (Huo et al., 2015). However, the direct immobilization of enzymes in ZIFs for size-selective catalysis in organic solvents has not been reported so far, which is very important for the controllable conversion of substrates with the same reactive groups.

In this work, we present the entrapment of enzyme CalB in ZIF-8 through biomimetic mineralization under room temperature, obtaining the hybrid material CalB@ZIF. Its application in size-selective biocatalysis for organic synthesis was investigated for the first time. In this hybrid CalB@ZIF, the ZIF-8 shell can not only enhance the activity and stability of CalB but also regulate the accessibility of substrates to the interior CalB.

## Materials and Methods

### Materials and Chemicals

*Candida antarctica* lipase B was purchased from c-LEcta GmbH (Leipzig, Germany), and the weight percentage of pure CalB in the received product is about 10% based on the Bradford test. Zn(NO_3_)_2_⋅6H_2_O, ethylenediaminetetraacetic disodium salt (EDTA-2Na), and Bradford test reagent Roti-Nanoquant were purchased from Carl Roth GmbH & Co. KG (Karlsruhe, Germany). Vinyl acetate, vinyl laurate, 2-methylimidazole, 1-butanol, and 3-(4-hydroxyphenyl)propan-1-ol were purchased from ABCR GmbH & Co. KG. Fluorescein isothiocyanate (FITC) was purchased from Sigma-Aldrich. Unless otherwise noted, all the chemicals were used as received without purification.

### Characterizations

Bradford test was performed on TECAN infinite M200. Thermogravimetric analysis (TGA) was carried out on NETZSCH STA 449F3 (NETZSCH, Germany) by heating samples from 25 to 800°C in a dynamic Ar atmosphere with a heating rate of 10°C min^–1^. Fourier transform infrared spectroscopy (FTIR) spectra were recorded on an FTIR spectrometer Tensor II (Bruker) with an attenuated total reflectance (ATR) unit. Powder X-ray diffraction (PXRD) was performed on a PANalytical X’Pert Pro powder diffractometer with Bragg–Brentano geometry equipped with a Ge(111)-monochromator, a rotating sample stage, and a PIXcel detector, using Cu Kα_1_ radiation (λ = 154.06 pm). The data were collected in reflection mode using a divergence slit that kept the illuminated sample area constant. Optical and fluorescence microscopy images were recorded on Olympus Provis AX70. N_2_ sorption was measured at 77 K on BeiShiDe (3H-2000PS2). The pore diameter of ZIF-8 was calculated through the Horvath–Kawazoe (H-K) method due to its microporous feature, while the pore diameter of CalB@ZIF was calculated through the Barrett–Joyner–Halenda (BJH) method because of its mesoporous feature. The results were given by the measurement instrument directly. Gas chromatography (GC) analysis was performed on a Shimadzu GC2010 PLUS gas chromatograph equipped with a BPX5 column (25 m × 0.22 mm) using a flame ionization detector (FID).

### Preparations

#### Preparation of CalB@ZIF

CalB@ZIF was synthesized following a reported method with modification (Liang et al., 2015). CalB (200 mg) was dissolved in 400 ml Zn(NO_3_)_2_⋅6H_2_O aqueous solution (80 mM). After stirring for 10 min under room temperature, 400 ml 2-methylimidazole aqueous solution (320 mM) was added. The mixture was stirred for 30 min, and the precipitated white solid was isolated through centrifugation at 4°C. After washing thoroughly with deionized water, the white solid was lyophilized and stored at −20°C before use. The amount of encapsulated CalB was determined by the Bradford test. CalB@ZIF (24.3 mg) was first digested in 1 ml saturated EDTA-2Na aqueous solution. After a transparent solution was obtained, the solution was dialyzed in deionized water for 24 h and lyophilized. The residual solid was then dissolved in 4 ml deionized water and subjected to the Bradford test.

#### Preparation of ZIF-8

Zeolitic imidazolate framework-8 was prepared by mixing 400 ml Zn(NO_3_)_2_⋅6H_2_O aqueous solution (80 mM) and 400 ml 2-methylimidazole aqueous solution (320 mM). After stirring for 30 min under room temperature, the white solid was isolated through centrifugation and washed thoroughly with deionized water before being lyophilized.

### Catalysis

#### A Typical Procedure for Transesterification Reaction

To a glass vial containing catalyst (CalB@ZIF or ZIF-8, 20 mg) or free CalB (366 μg, the amount was determined through Bradford test), alcohol (300 mM) and vinyl ester (200 mM) in acetone (250 μl) were added. The sealed vial was shaken at 800 rpm under 25°C. At interval time, the reaction mixture (5 μl) was withdrawn and diluted to 100 μl with acetone. The solid catalyst was removed through centrifugation, and the obtained supernatant was analyzed by GC. The conversion was determined by the diminishment of the peak of vinyl ester.

#### Thermal Stability Measurement

CalB@ZIF (20 mg) or free CalB (366 μg, the amount was determined through Bradford test) was dispersed in 250 μl acetone in glass vials. The sealed vials were shaken at 800 rpm under 50°C. At intervals (0, 1, 2, 4, and 8 h), the glass vials were removed from heating. After cooling to room temperature, n-butanol (5.56 mg, 0.075 mmol) and vinyl acetate (4.30 mg, 0.050 mmol) were added. After being shaken at 800 rpm under 25°C for 25 min, 5 μl reaction mixture was withdrawn and diluted to 100 μl. The supernatant was subjected to GC analysis.

#### Reusability of CalB@ZIF

To a glass vial containing 20 mg CalB@ZIF, n-butanol (300 mM) and vinyl acetate (200 mM) in acetone (250 μl) were added. The sealed vial was shaken at 800 rpm under 25°C for 2 h. After isolating the solid catalyst from the reaction mixture, the supernatant was analyzed with GC. The isolated CalB@ZIF was washed with acetone (3 × 1 ml) and dried in a vacuum under room temperature before being used in the next run.

## Results and Discussion

**SCHEME 1:**
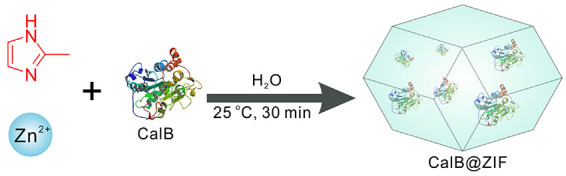
Schematic illustration of the synthesis of CalB@ZIF.

CalB@ZIF was prepared by mixing an aqueous solution of Zn(NO_3_)_2_⋅6H_2_O containing CalB and an aqueous solution of 2-methylimidazole, following a reported method with modification (Liang et al., 2015). The schematic illustration of the synthesis of CalB@ZIF is depicted in Scheme 1. ZIF-8 was prepared under identical reaction conditions but in the absence of CalB. It should be mentioned that the formation of ZIF-8 is much slower compared with the formation of CalB@ZIF, indicating that the nucleation of ZIF-8 precursors can be triggered by CalB (Maddigan et al., 2018). The content of trapped CalB in CalB@ZIF was determined based on a standard Bradford assay after digesting CalB@ZIF in EDTA-2Na solution, which turned out to be 18.3 μg mg^–1^.

**FIGURE 1:**
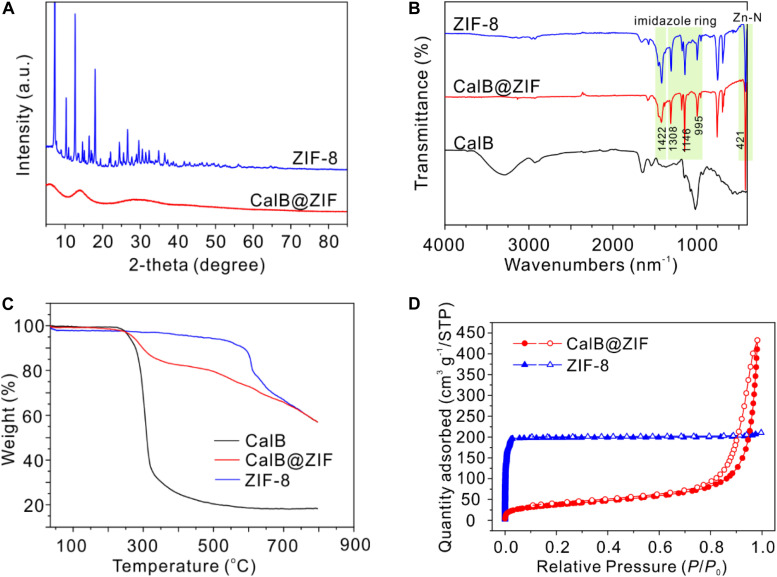
**(A)** Powder X-ray diffraction (PXRD) patterns of CalB@ZIF and ZIF-8. **(B)** Fourier transform infrared spectroscopy (FTIR) spectra of ZIF-8, CalB@ZIF, and CalB. **(C)** Thermogravimetric analysis (TGA) curves of ZIF-8, CalB@ZIF, and CalB. **(D)** N_2_ sorption isotherms of ZIF-8 and CalB@ZIF.

Powder X-ray diffraction measurement was performed to characterize the crystallinity of CalB@ZIF (Figure 1A). Unexpectedly, no characteristic peaks from ZIF-8 are observed in the PXRD pattern of CalB@ZIF, indicating the formation of amorphous ZIF-8. Amorphous MOFs (aMOFs) are an emerging family of MOF materials, which maintain the basic building blocks but without long-range crystallinity (Bennett and Cheetham, 2014; Bennett and Horike, 2018). Preparation of aMOFs usually requires harsh conditions, including high-pressure treatment (Chapman et al., 2009, 2011), high-temperature heating (Bennett et al., 2011), electron-beam treatment (Conrad et al., 2018), and high-energy ball-milling (Cao et al., 2012). While in our case, the amorphization of ZIF-8 is realized through a biomolecule-induced process in a mild condition. The amorphous feature of CalB@ZIF is probably caused by the coordination between amine groups of CalB and Zn^2+^, whereby CalB functions as a competitive ligand against 2-methylimidazole and disturbs the periodic structure of ZIF-8 (Ge et al., 2012; Liu et al., 2020).

The formation of ZIF-8 structure in CalB@ZIF is confirmed by FTIR measurement (Figure 1B). In the FTIR spectrum of CalB@ZIF, the adsorption bands, which can be assigned to ZIF-8 structure, are clearly observed. The sharp signal at 421 cm^–1^ is attributed to Zn-N stretching, while the bands at 1,422 and 900–1,300 cm^–1^ are derived from the stretching and plane bending of the imidazole ring, respectively (Hou et al., 2015). The signal from CalB in CalB@ZIF is almost invisible probably due to the low amount of trapped CalB. TGA results confirm the presence of CalB in CalB@ZIF (Figure 1C). The first-stage weight loss of CalB@ZIF starts from around 250°C due to the decomposition of CalB within the framework, while pure ZIF-8 starts to decompose at nearly 450°C. It is noted that the thermal stability of ZIF-8 decreases slightly in CalB@ZIF, which is possible because of its amorphous structure in the hybrid material.

The porosity of CalB@ZIF and pure ZIF-8 was investigated with the N_2_ adsorption/desorption experiment (Figure 1D). The isotherm of ZIF-8 displays a steep increase at low relative pressure (*P*/*P*_0_ < 0.1), indicating the existence of permanent micropores. And the average pore diameter of ZIF-8 is 0.799 nm calculated through the H-K method. On the contrary, a sharp rise at high relative pressure in the N_2_ adsorption isotherm of CalB@ZIF is observed, suggesting the presence of interparticle mesopores. The average pore diameter of CalB@ZIF is 18.380 nm calculated through the BJH method. Meanwhile, the appearance of hysteresis demonstrates the deformation and swelling of CalB@ZIF (Weber et al., 2010). The discrepancy between the N_2_ adsorption properties of CalB@ZIF and ZIF-8 is possible because of the presence of CalB and CalB-induced amorphization of ZIF-8, which may affect its pore structures (Chapman et al., 2009).

**FIGURE 2:**
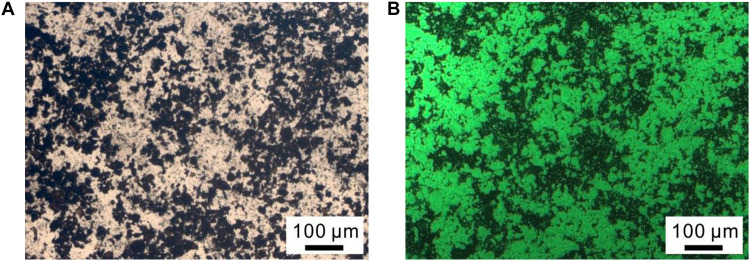
**(A)** Optical and **(B)** fluorescent microscopy images of CalB-FITC@ZIF.

To further confirm the presence of CalB in CalB@ZIF, CalB labeled with FITC (CalB-FITC) was embedded in ZIF-8 following the same procedure for preparing CalB@ZIF, and the obtained sample was denoted as CalB-FITC@ZIF. The black particles observed on the optical microscopic image are the sample CalB-FITC@ZIF (Figure 2A). These particles emit green fluorescence when observed under a fluorescence microscope (Figure 2B). In a control experiment, no fluorescence is emitted from ZIF-8 particles (Supplementary Figure 1). These results manifest the successful entrapment of enzymes in CalB@ZIF.

**FIGURE 3:**
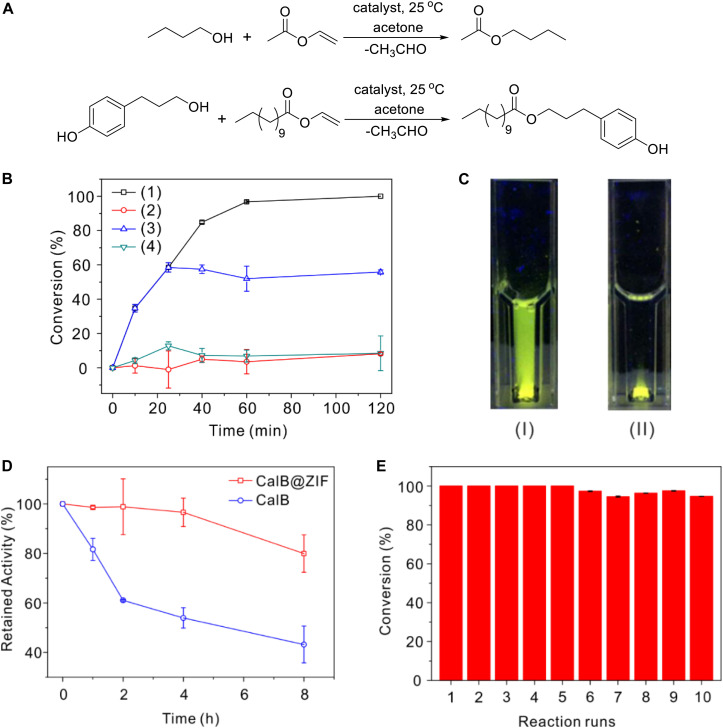
**(A)** Reaction schemes of the model reactions. **(B)** Time-dependent conversion of vinyl acetate or vinyl laurate: (1) conversion of vinyl acetate when CalB@ZIF is used, (2) conversion of vinyl laurate when CalB@ZIF-8 is used, (3) conversion of vinyl acetate in the leaching test (CalB@ZIF was isolated at 30 min), and (4) conversion of vinyl acetate in the presence of ZIF-8 instead of CalB@ZIF. **(C)** Photo picture of CalB-FITC@ZIF (I) and CalB-FITC (II) in acetone observed under a UV light (245 nm). **(D)** Thermal stability test of CalB@ZIF and free CalB. **(E)** Reusability test of CalB@ZIF.

*Candida antarctica* lipase B is a widely used broad-substrate lipase for esterification and transesterification reactions. The catalytic performance of CalB@ZIF was assessed by carrying out transesterification reactions between a pair of small substrates (butanol and vinyl acetate) and a pair of larger ones [3-(4-hydroxyphenyl)propan-1-ol and vinyl laurate] in acetone (Figure 3A; Huo et al., 2015). The reactions were carried out under room temperature, and the conversion was monitored by GC. Initially, the reactions were performed with free CalB as the catalyst. After 120 min, moderate conversion of vinyl acetate and vinyl laurate was found, which was 51.4 and 64.7%, respectively (Supplementary Figure 2). When CalB@ZIF was used as a catalyst, it was found that vinyl acetate was almost totally converted within 120 min using the small substrates (Figure 3B). However, the conversion of vinyl laurate was only 8.2% using the large substrates. In a control experiment, it was also found that 8.5% of vinyl acetate was converted in the presence of ZIF-8 instead of CalB@ZIF. These results reveal that the smaller substrates and their products can readily diffuse through the apertures of ZIF-8, while larger substrates cannot pass through the pores. Leaching test was carried out by isolating CalB@ZIF from the reaction mixture after reacting 30 min, and almost no further conversion of vinyl acetate was observed, indicating that no CalB leached out from CalB@ZIF during the catalysis. It is noteworthy that CalB@ZIF shows a higher catalytic activity than free CalB in catalyzing the transesterification reaction. The enhanced activity of CalB in CalB@ZIF is presumably derived from the conformational change of CalB induced by coordination between CalB and Zn^2+^, which is called allosteric effect (Ge et al., 2012; Wang et al., 2013). Meanwhile, the microenvironments created by ZIF-8 shell may also contribute to the enhanced activity of encapsulated CalB (Lyu et al., 2014; Sun et al., 2018). Additionally, CalB@ZIF has a much better dispersibility than free CalB in acetone, which is very important to the higher catalytic activity as well. As shown in Figure 3C, when CalB-FITC@ZIF is added into acetone and observed under a UV light (245 nm), the whole mixture emits green fluorescence due to the good dispersibility of CalB-FITC@ZIF. But when CalB-FITC is added into acetone, green fluorescence is only observed at the bottom part. The aggregation of free CalB restricts its catalytic ability in organic solvents (Stepankova et al., 2013).

The encapsulation of CalB with ZIF-8 not only offers the catalyst size selectivity and enhanced activity but also elevates the thermal stability of CalB. When CalB@ZIF was used to catalyze the transesterification between vinyl acetate and n-butanol after being incubated in acetone under 50°C for 8 h, it was found that the conversion of vinyl acetate was still higher than 80% of that catalyzed with untreated CalB@ZIF (Figure 3D). Yet the retained activity of free CalB decreased to only about 50%. These results illustrate the high thermal stability of CalB encapsulated in ZIF shells and their potential as a robust catalyst for industry-relevant application in organic solvents. Reusability is also a crucial parameter when catalysts are used in the industry. The reaction between vinyl acetate and n-butanol was still used as the model reaction. As shown in Figure 3E, only a slight decrease in conversion is observed even after CalB@ZIF is used for 10 times. The high reusability of CalB@ZIF is probably because the conformational change of CalB caused by acetone is inhibited by the ZIF shells.

## Conclusion

In conclusion, a facile method for the direct encapsulation of the enzyme CalB in ZIF-8 is reported. Interestingly, amorphous ZIF-8 shell forms during the preparation, which is probably induced by the coordination between CalB and Zn^2+^. Size-selective biocatalysis properties of the hybrid CalB@ZIF is investigated for the first time through catalyzing transesterification reaction in organic solvent. The embedded CalB also shows enhanced activity and thermal stability. This work sheds light on the possibility to fabricate enzyme-MOF hybrid catalysts in a straightforward way for task-specific and long-term usage in industrial applications.

## Data Availability Statement

All datasets generated for this study are included in the article/Supplementary Material.

## Author Contributions

YW and CW designed this project. YW performed most of the experiments and wrote the main part of the manuscript. CW coordinated the research and corrected and revised the manuscript. NZ and DT carried out some characterization experiments. ZQ offered constructive suggestions for this research and was responsible for the final correction and English proofing. All authors contributed to the article and approved the submitted version.

## Conflict of Interest

The authors declare that the research was conducted in the absence of any commercial or financial relationships that could be construed as a potential conflict of interest.
