# Supplementary material for: Facile Synthesis of Enzyme-Embedded Metal–Organic Frameworks for Size-Selective Biocatalysis in Organic Solvent
Source: Front Bioeng Biotechnol. 2020 Jul 7;8:714. doi: 10.3389/fbioe.2020.00714 (PMC7358279; doi:10.3389/fbioe.2020.00714)
Supplement: Supplementary file 1 [file Image_1.pdf]

Supplementary Material

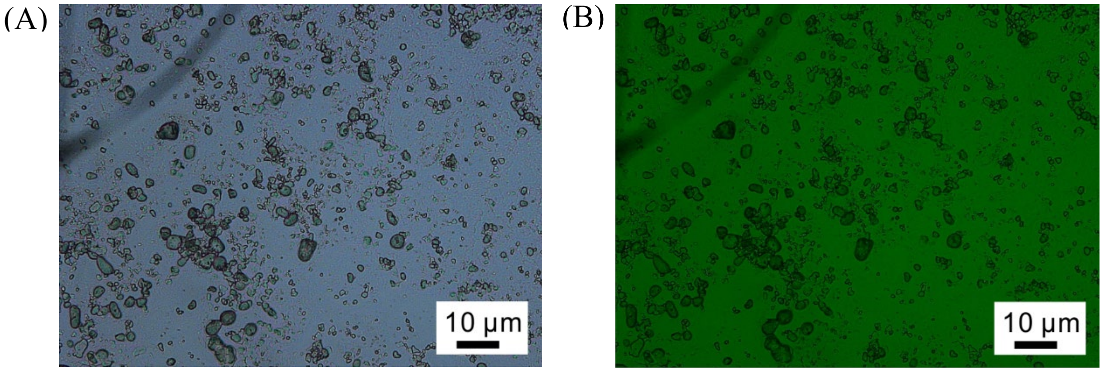

Supplementary Figure 1. (A) Optical and (B) fluorescent microscopy images of ZIF-8. The black particles in (B) are ZIF-8

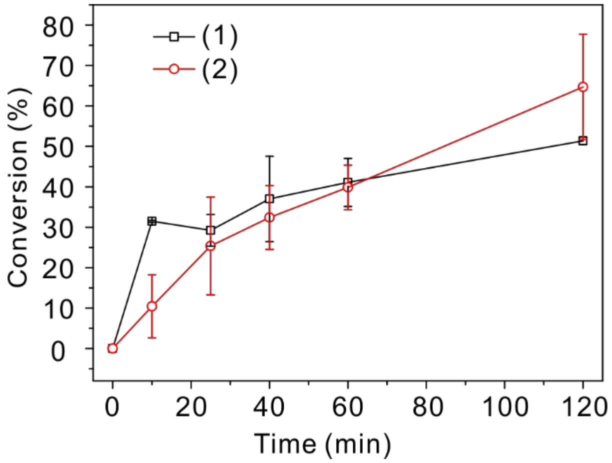

Supplementary Figure 2. Conversion of (1) vinyl acetate and (2) vinyl laurate when free CalB was used as the catalyst. Reaction conditions: n-butanol or 3-(4-hydroxyphenyl)pronan-1-ol (300 mM), vinyl acetate or vinyl laurate (200 mM), free CalB (366 μg, determined through Bradford test), acetone (250 μL), 25 ºC.
